# Male patient with mild cognitive impairment and extremely high P300 and Slow-wave latencies: a case report

**DOI:** 10.4076/1757-1626-2-6157

**Published:** 2009-07-01

**Authors:** Vasileios T Papaliagkas, Magda N Tsolaki, Vasileios K Kimiskidis, Georgios Anogianakis

**Affiliations:** 1 Department of Experimental Physiology, Aristotle University of Thessaloniki, Thessaloniki, Greece; 2 Third Department of Neurology, Aristotle University of Thessaloniki, "G. Papanikolaou" Hospital, Thessaloniki, Greece

## Abstract

We present a case of a 74-year-old Greek male who suffered from paraphasias, memory and orientation problems. The patient was assessed with neuropsychometric tests, auditory event-related potentials and cerebrospinal fluid proteins and was diagnosed with mild cognitive impairment. The emphasis on the case is on the unexplained high levels of P300 and Slow wave of the auditory event-related potentials.

P300 is believed to be delayed in Alzheimer's Disease (AD), however in our case it was extremely prolonged in baseline and follow-up examinations without AD being diagnosed. This might suggest that AD is a complex and multifactorial disease.

## Case presentation

A 74-year-old Greek male patient was referred to the Memory outpatients clinic of the "G. Papanikolaou" Hospital in 7 February 2005 due to paraphasias, memory and orientation problems. The orientation problems were reported to be worse at night. The patient was 1.78 meters tall and 95 kg and had 6 years of education. His problems began 7 years before with initial symptoms mood and behavioural changes. One initiating factor that was mentioned was the loss of his brother.

From his past medical history the patient underwent prostate removal in 2001 and suffered from back pain. Moreover, the patient had high blood cholesterol levels for which he was receiving drug therapy from 2004 (Lipitor 40 mg 1 × 1). He was also taking Salospir 100 mg S = 1 × 1. No family history for AD or other form of dementia was reported.

Neuropsychological examination was performed. The score in the Mini-Mental State Examination (MMSE) [1,2] scale was 27/30 and the Clinical Dementia Rating (CDR) [3] scale was 0.5 suggesting that global cognitive function is satisfactory. The score in the Geriatric Depression Scale (GDS) [4] was 0/15, excluding depression as a diagnosis.

Neuroimaging studies (MRI) revealed slight microdegenerative changes of arterial type and slight atrophy of the right hippocampus. The patient was diagnosed as having Mild cognitive impairment (MCI).

Auditory event-related potentials were performed to the patient, who was included in the study of Papaliagkas et al. [5]. The P300 and SW latencies were found to be 591 ms and 779 ms respectively. Compared to the mean values of the latencies observed in a group of 91 MCI patients (mean ±SD value for P300: 406.4 ± 51.8 ms and for SW: 536.35 ± 62.11 ms) the patient's figures were higher by more than 3 standard deviations for P300 and by more than 4 for SW. On the contrary, the value of N200 latency (260 ms) was approximately equal to the respective mean value of the MCI patients (252 ms). A CSF sample was also obtained with lumbar puncture. The β-amyloid_(1-42)_ and tau levels were determined, using the sandwich ELISA INNOTEST β-amyloid_(1-42)_ and hTau-Antigen sandwich ELISA kits of Innogenetics, Ghent, Belgium. Both protein levels were within normal levels according to the kit manufacturer (β-amyloid_(1-42)_ = 911 pg/ml, tau = 194 pg/ml). A follow-up examination was performed after 12 months. MMSE score was stable (27/30). P300 and SW latencies continued to be extremely high (P300 = 625 ms and SW = 751 ms), whereas N200 latency (268 ms) was still approximately equal to the mean value of the MCI patients (255 ms). Furthermore, CSF proteins continued to be within normal levels (β-amyloid_(1-42)_ = 791 pg/ml, tau = 109 pg/ml).

## Discussion

Cognitive event-related potentials (ERPs) have been widely used in the study of dementias, including Alzheimer's disease. P300 component corresponds to mental processes such as recognition, categorization of stimuli, expectancy or short-term memory, while there are many regions in the brain, especially in the temporal lobe, the parietal lobe and the hippocampus which are thought to be responsible for its generation [6].

The numerous clinical P300 studies [6]-[11], strongly suggest that it may be clinically useful as an index of cognitive function and that it is prolonged in AD patients. N200 and P300 latencies were found to be significantly prolonged in AD patients when compared to either MCI patients or controls and this difference might help categorize patients into one of the three groups [7].

In our case study it is interesting to note that although the patient's P300 and SW latencies were extremely high, he did not suffer from AD. This increase might be due to the microdegenerative changes of arterial type and the atrophy of right hippocampus, however it cannot be verified if these slight changes can explain such extremely high latencies. P300 latency may increase slightly in minor ischemic stroke and this increase is associated with post-stroke depression [12], which was not diagnosed in our patient. No increased P300 latency has been observed in patients with vascular cognitive impairment [13]. It is also interesting to note that the high values of P300 and SW latencies were not accompanied by an increase in N200 latency, which implies that the mechanism of production of the N200 wave is independent of that of the two sequential waves.

## Abbreviations

AD: Alzheimer's disease; CDR: Clinical Dementia Rating; CSF: Cerebrospinal fluid; ERP: Event-related potentials; MCI: Mild cognitive impairment; MMSE: Mini-Mental State Examination; MRI: Magnetic Resonance Imaging; SW: Slow wave.

## Consent

Written informed consent was obtained from the patient for publication of this case report. A copy of the written consent is available for review by the Editor-in-Chief of this journal.

## Competing interests

The authors declare that they have no competing interests.

## Authors' contributions

VTP contributed to the design of the study, carried out the event-related potentials, performed the statistical analysis and drafted the manuscript. VKK contributed to the design of the study, the event-related potentials and the manuscript preparation. MNT recruited the patients and the control subjects and provided the neurological diagnosis and interpretation. GAA contributed to the design and coordination of the study and the manuscript preparation. All authors read and approved the final manuscript.
